# Effect of standardized training in combination with masseter sensitization on corticomotor excitability in bruxer and control individuals: a proof of concept study

**DOI:** 10.1038/s41598-022-21504-w

**Published:** 2022-10-19

**Authors:** Noéli Boscato, Fernando G. Exposto, Yuri M. Costa, Peter Svensson

**Affiliations:** 1 Department of Restorative Dentistry, School of Dentistry, Federal University of Pelotas, Pelotas, Rio Grande do Sul Brazil; 2 Section for Orofacial Pain and Jaw Function, Department of Dentistry and Oral Health, Aarhus University, Aarhus, Denmark; 3 Scandinavian Center for Orofacial Neurosciences (SCON), Aarhus, Denmark; 4 Department of Biosciences, Piracicaba Dental School, University of Campinas, Piracicaba, Brazil; 5 Faculty of Odontology, Malmö University, Malmö, Sweden

**Keywords:** Neuroscience, Physiology, Neurology, Signs and symptoms

## Abstract

Recently, it has been proposed that bruxism could represent an overlearned behavior due to the absence of corticomotor plasticity following a relevant tooth-clenching task (TCT). This study assessed the modulatory effects of a nerve growth factor (NGF) injection on masseter muscle corticomotor excitability, jaw motor performance, pain, and limitation in bruxer and control participants following a TCT. Participants characterized as definitive bruxers or controls were randomly assigned to have injected into the right masseter muscle either NGF or isotonic saline (IS), resulting in a study with 4 arms: IS_Control (n = 7), IS_Bruxer (n = 7); NGF_Control (n = 6), and NGF_Bruxer (n = 8). The primary outcome was the masseter motor evoked potential (MEP) amplitude measured at baseline and after a TCT. After the interventions, significantly higher and lower MEP amplitude and corticomotor map area were observed, respectively, in the IS_Control and NGF_Control groups (*P* < 0.05). Precision and accuracy depended on the series and target force level with significant between-group differences (*P* < 0.01). NGF‐induced masseter muscle sensitization, in combination with a training-induced effect, can significantly impact the corticomotor excitability of the masseter muscle in control participants indicating substantial changes in corticomotor excitability, which are not observed in bruxers. These preliminary findings may have therapeuthic implications for the potential to “detrain” and manage bruxism, but further studies with larger sample sizes will be needed to test this new concept.

## Introduction

The scientific evidence is conflicting and does not sufficiently support a direct linear causal relationship between bruxism and orofacial pain, although bruxism, to some extent, has been associated with musculoskeletal symptoms^1,2^. Bruxism is characterized by repetitive jaw-muscle activity during awake or sleep periods that can be noticed as clenching or grinding of the teeth and/or by bracing or thrusting the mandible^3^. Clinical studies have shown that bruxers with low frequency of electromyographic (EMG) jaw-muscle activity tend to report craniofacial pain more often than those with higher EMG activity. Probably, pain may affect the motor response and decrease EMG jaw-muscle activity to protect the affected region and reduce pain^4^, although the mechanisms underlying the relationship between pain and motor function are not yet fully established^5,6^. For instance, the so-called vicious cycle theory^7^ takes into account a simplistic cause-effect view, such as the assumption that jaw-muscle hyperactivity leads to more pain due to an overloaded masticatory system; however, such relationships are not well supported by scientific evidence and should be viewed with caution^8,9^.

Previous studies have shown that intramuscular administration of nerve growth factor (NGF), by inducing masseter muscle soreness and pain in jaw function, can significantly reduce jaw muscle corticomotor excitability, decreasing motor-evoked potential (MEP) in healthy individuals^5,10^. Another study showed that a standardized tooth clenching task (TCT) is associated with increased cortical neuroplasticity in non-bruxers, but not in bruxers^11^, suggesting that repeated jaw movements could trigger neuroplastic changes in the corticomotor control of jaw-closing muscles in healthy individuals. As such, the authors raised the possibility that bruxism is an overlearned condition; therefore, bruxers would need to unlearn it^11^. Since the available scientific evidence strongly suggests that a more detailed assessment of the causal relationship between musculoskeletal orofacial pain and jaw motor activity should be obtained, it is important to understand if muscle pain induced by NGF-induced sensitization can create new neural jaw muscle activity pathways and/or modify existing ones. While experimental models associated with longer‐lasting muscle sensitization, e.g., intramuscular administration of NGF^5,10^, and tooth clenching behavior^11^ are suitable to evaluate motor neuroplasticity related to muscle pain and jaw-closing muscles performance, the relationship between central modulation of motor activity in response to NGF-induced sensitization can, indeed, be elucidated by the quantification of MEPs with the aid of transcranial magnetic stimulation (TMS)^12–14^. Human experimental muscle pain models are regarded as the bridge between basic and clinical pain research, and, therefore, can help in translating mechanistic knowledge into clinical practic ^5,10^.

Therefore, this study aimed to compare MEP amplitude, jaw movement performance (i.e., accuracy and precision of maximum voluntary contraction [MVC]), pain intensity, and limitation in participants classified as bruxers or controls that received either NGF or isotonic saline (IS) before a standardized TCT. Taking into consideration previous evidence that indicates reduced central modulation of motor activity in response to experimental muscle pain^5,10^, and distinct pathways of cortical neuroplasticity between bruxers and controls^11^, we hypothesized a priori that, after 58-min of a standardized training, NGF-induced sensitization would provoke a significant decrease in the corticomotor excitability in controls, but not in bruxers.

## Results

### MEP amplitude

**Figure 1:**
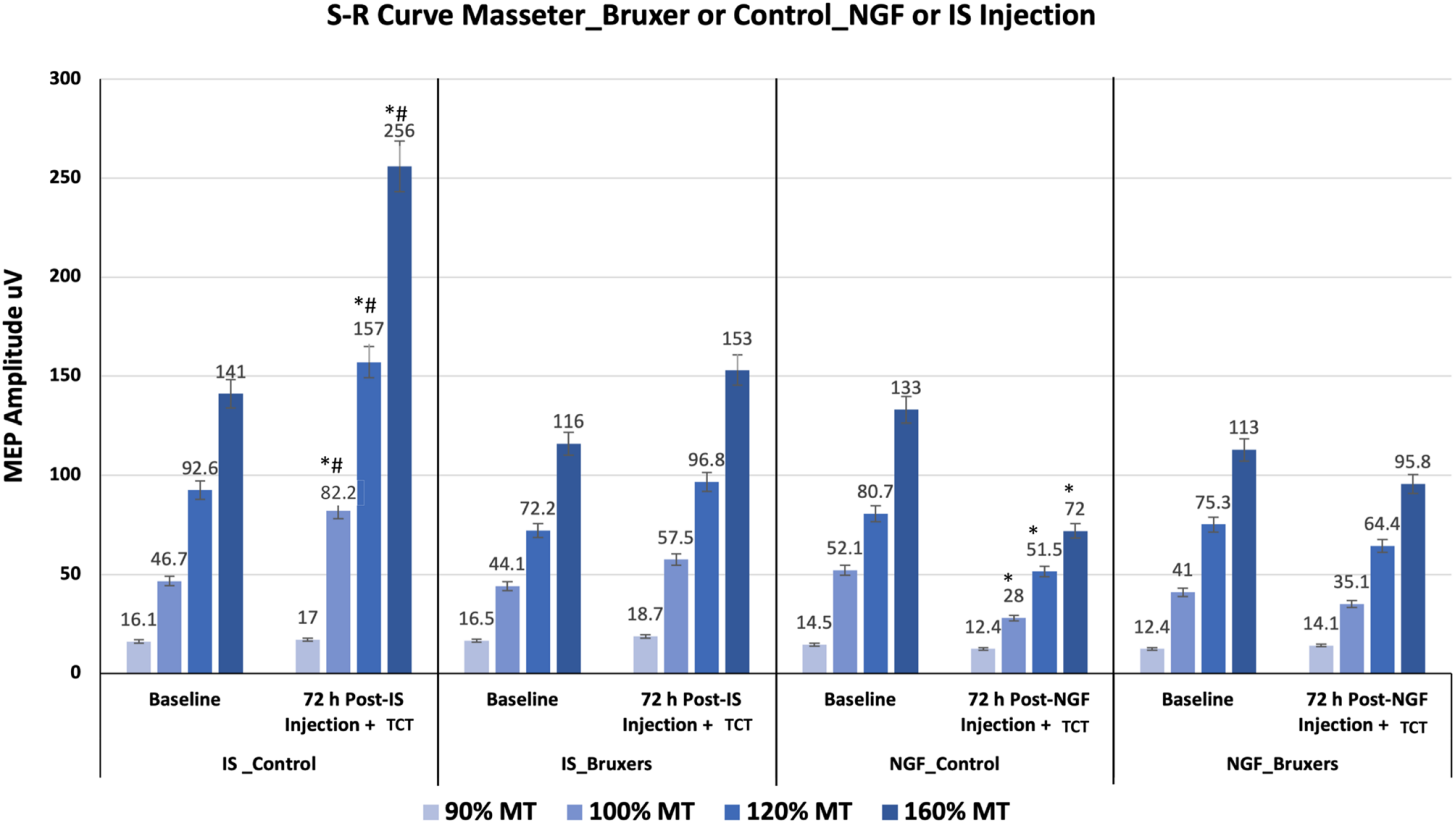
S–R curves from masseter MEP elicited by TMS at two-time points: baseline and 72 h post-injection and a TCT. (*) Indicates significant higher (IS_Control) and lower (NGF_Control) MEP amplitude within-group differences comparing values (mean and SEM) at baseline and post-injection and TCT (*P* < 0.05). There were no significant MEP amplitude within-group differences for bruxer participants (IS_Bruxer and NGF_Bruxer groups) regardless they had received IS or NGF (*P* > 0.05). (#) indicates significant higher MEP amplitude differences between‐group (Intensity, at 90, 100, 120 and 160% MT), *(P* < 0.05). Tooth clenching task (TCT); stimulus–response (S–R) curves; motor threshold (MT); motor evoked potential (MEP); standard error (SE); transcranial magnetic stimulation (TMS); nerve growth factor (NGF); isotonic saline (IS).

There were no significant differences between bruxer and control participants in the masseter MEP amplitude at baseline (*P* = 0.378). Masseter MEP amplitudes were significantly dependent on group (i.e., control or bruxer participants) (*P* < 0.05), stimulus intensity (*P* < 0.05) and assessment time (*P* < 0.05). Overall the participants who received the NGF injection had decreased MEP amplitude 72 h post-injection and a TCT, while those who received IS had increased MEP amplitude, with significantly higher and lower MEP values revealed, respectively, in the IS_Control and in the NGF_Control group at 100, 120, 160% MT (*P* < 0.05). In contrast, there were no significant differences in the masseter MEP amplitude at baseline and post-injection and TCT in the bruxers (NGF_Bruxer and IS_Bruxer), regardless of the injection received (*P* > 0.05) (Fig. 1). Indeed, there was a significant effect of stimulation intensity on the MEP amplitude in all groups with higher MEPs at higher intensities; while the highest MEP amplitude values were observed at 160% MT in the IS_Control group (*P* < 0.01).

**Figure 2:**
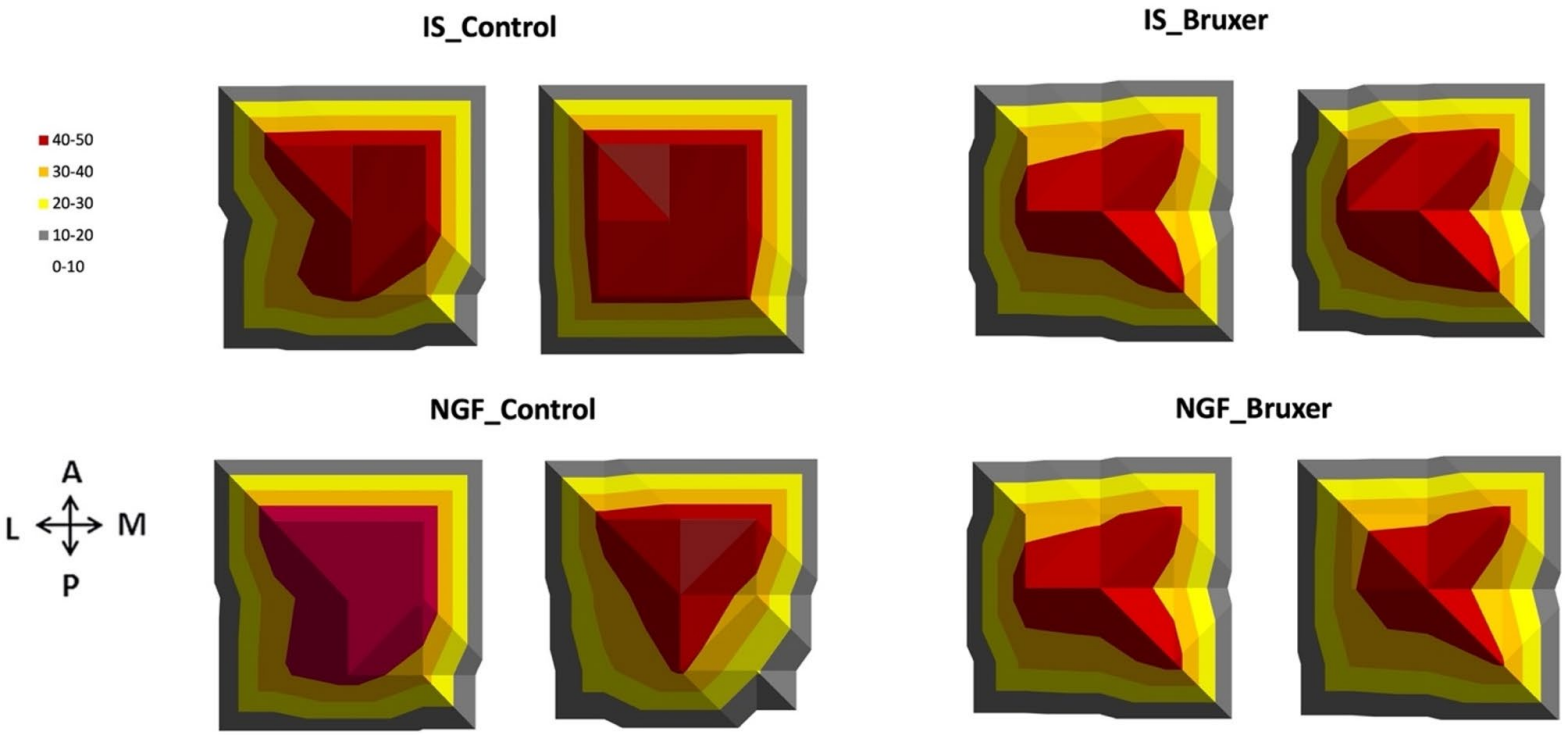
Masseter MEP corticomotor map area elicited by TMS of multiple scalp sites arranged in a one × 1 cm^2^ grid at 120% MT before (baseline) and 72 h after (post‐injection) the intramuscular administration of NGF and IS in the bruxer and control participants. Arrows indicate directions (anterior, posterior, medial, and lateral). Motor threshold (MT); motor evoked potential (MEP); nerve growth factor (NGF); isotonic saline (IS); transcranial magnetic stimulation (TMS).

In the FDI, the control hand muscle, the MEPs values were dependent on stimulus intensity (*P* < 0.01) but not on the group and assessment time**.** Likewise, there was a significant main effect of the injection on the masseter MEP map area in control participants, where the NGF_Control group presented smaller, and the IS_Control greater masseter MEP corticomotor map areas (i.e., ≥ 50% max) after the TCT (*P* < 0.01), while no significant map area differences were identified in the bruxers (Fig. 2).

### Maximum voluntary contraction (MVC)

**Figure 3:**
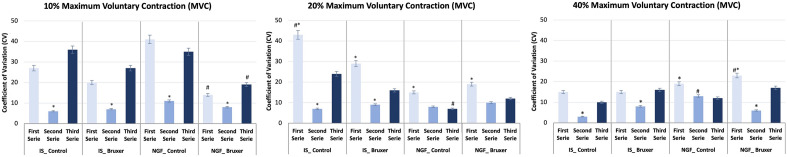
Illustrates the precision of MVC 72 h after the intramuscular administration of either IS or NGF considering differences in the jaw movement performance between actual force values and target force levels at 10, 20, and 40% MVC in the bruxers that received IS or NGF (IS_Bruxer; NGF_Bruxer); and controls that received IS or NGF (IS_Control; NGF_Control). Precision is expressed as the coefficient of variation (CV) of actual force level at each force level and series. (*) Indicates significant within-groups and (#) significant between-groups differences at 10, 20, and 40% MVC considering the first, second, third series, and target force level following pairwise post hoc comparisons (*P* < 0.01). Tooth clenching task (TCT); maximum voluntary contraction (MVC); nerve growth factor (NGF); isotonic saline (IS).

Precision and accuracy of jaw movements were significantly dependent on the series and target force level with significant between-group differences (*P* < 0.01). Overall, the precision in the second series was significantly higher (i.e., coefficient of variation (CV) was lower) than in the first and third series for all groups (*P* < 0.01). There was also a significant CV decrease in the first to the third series for all groups at 20% MVC, and in the NGF_Control and NGF_Bruxer at 40% MVC (*P* < 0.01). Significant between-group differences were observed, with the NGF_Bruxer group showing a significantly lower CV (i.e., higher precision MVC) at 10% in the first and third series. At 20% MVC, the IS_Control showed significantly higher CV (i.e., lower precision) in the first series, and the NGF_Control lower CV (i.e., higher precision) in the third series (*P* < 0.01). Finally, at 40% MVC, the NGF_Bruxer indicated a higher CV in the first series and the NGF_Control in the second series (*P* < 0.01) (Fig. 3).

**Figure 4:**
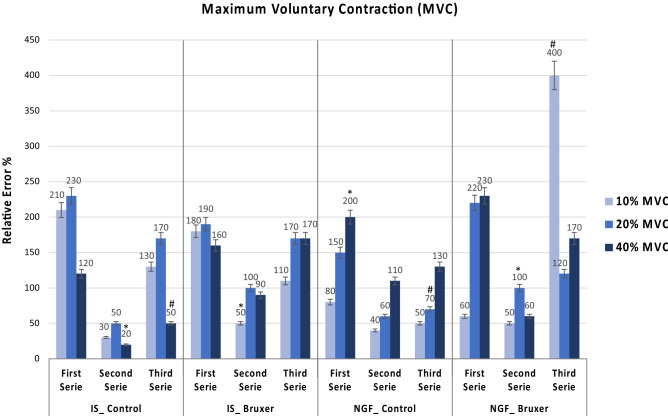
Illustrates the accuracy of the MVC 72 h after the intramuscular administration of either IS or NGF considering differences in the jaw movement performance between actual force values and target force levels at 10, 20, and 40% MVC in the bruxers that received IS or NGF (IS_Bruxer; NGF_Bruxer); and controls that received IS or NGF (IS_Control; NGF_Control). Accuracy is expressed as the relative error (RE) of the actual force level at each force level and series. (*) Indicates significant within-groups and (#) significant between-groups differences at 10%, 20%, and 40% MVC considering first second and third series, and target force level following pairwise post hoc comparisons (*P* < 0.01). Tooth clenching task (TCT); maximum voluntary contraction (MVC); nerve growth factor (NGF); isotonic saline (IS).

Considering within-group differences, significantly higher accuracy (i.e., the relative error was smaller) was found in the second series than in the first and third series at 10, 20, and 40% MVCs for IS_Bruxer, IS_Control, and NGF_Bruxer, but not for NGF_Control (*P* < 0.05). In the third series, significant between-group differences were revealed, with the NGF_Bruxer showing significantly lower accuracy (i.e., the relative error was higher) at 10% MVC (Bonferroni: *P* < 0.01). In contrast, the NGF_Control at 20% MVC and the IS_Control at 40% MVC showed significantly higher accuracy in the third series (i.e., the relative error was lower) (*P* < 0.01) (Fig. 4).

### Electromyographic (EMG) recordings

There were significant between-group differences in the EMG events/hour during sleep, with the higher number of EMG events/hour observed in the bruxer group: NGF_Bruxer (30.0 ± 7.5) and IS_Bruxer (28.0 ± 8.9), and the lower in the control participants: NGF_Control (13.7 ± 3.6) and IS_Control (14.4 ± 4.5) (*P* < 0.01).

Between-group comparison of EMG CV values considering baseline and post-injection revealed significant differences, in which the NGF_Bruxer participants showed significantly higher CV values (21.0%) compared with the other groups, and the NGF_Control the lower CV values (2.0%) (*P* < 0.01). Among the 15 participants characterized as bruxers by EMG recordings, 11 also self-reported grinding their teeth or clenching their jaw while sleeping at night, and just 1 participant reported “pain or stiffness in your jaw on awakening,” and 1 “pain in your jaw from jaw habits or activity.” A significant between-group difference in the EMG events/hour during sleep with a higher number of EMG events/hour was observed in the participants who self-reported possible bruxism (*P* = 0.004).

Finally, the assessment of jaw pain symptoms and function, psychological and sleep conditions showed that after NGF-sensitization, higher scores of jaw pain on chewing, fatigue, jaw function limitation, and chewing ability, and also, decreased pain‐free mouth open in the bruxer and control participants were observed, with between-group differences revealed in the NGF_Control and NGF_Bruxer groups (*P* = 0.002). In contrast, IS injection did not cause significant jaw pain or jaw function disturbance (*P* > 0.05). Indeed, there were no significant between‐group differences considering the GAD-7 (*P* = 0.337), PSQI (0.665), OBC (*P* = 0.791) and PHQ–9 (*P* = 0.245) scores at baseline (Table 1). Finally, higher perceived chewing ability scores were identified at baseline in those who self-reported possible bruxism than those that did not report (*P* = 0.009).

**Table 1:** Clinical assessment of jaw pain and function and psychological and sleep conditions at baseline and 72 h after the intramuscular administration of either isotonic saline (IS) or nerve growth factor (NGF).

Outcomes	IS_control (n = 7)		IS_bruxer (n = 7)		NGF_control (n = 6)		NGF-bruxer (n = 8)	
	Baseline	72 h post-injection	Baseline	72 h post-injection	Baseline	72 h post-injection	Baseline	72 h post-injection
Jaw pain at rest (0–10 NRS)	0.0 (0.0)	0.0 (0.0)	0.0 (0.0)	0.0 (0.0)	0.0 (0.0)	0.2 (0.2)	0.0 (0.0)	0.4 (0.7)
Jaw pain on chewing (0–10 NRS)	0.0 (0.0)	0.0 (0.0)	0.0 (0.0)	0.0 (0.0)	0.0 (0.0)	1.0 (0.2)*	0.0 (0.0)	0.8 (0.5)*
Jaw fatigue (0–10 NRS)	0.0 (0.0)	0.0 (0.0)	0.7 (0.7)	0.7 (0.3)	0.0 (0.0)	0.9 (0.6)*	0.0 (0.0)	0.6 (1.5)*
Pain‐free mouth opening (mm)	48.0 (1.6)	47.4 (2.1)	50.4 (1.5)	50.0 (1.2)	53.0 (5.1)	43.0 (5.0)*	48.0 (1.7)	35.0 (3.8)*#
JFLS–20	0.0 (0.0)	0.0 (0.0)	0.1 (0.0)	0.1 (0.3)	0.0 (0.1)	1.4 (0.3)*#	0.8 (0.5)	1.0 (0.7)
PCA, easy bite and chew foods	1.1 (0.1)	1.1 (0.1)	1.1 (0.1)	1.1 (0.1)	1.0 (0.0)	4.5 (0.7)*#	2.0 (0.6)	3.0 (0.7)*
PCA, satisfied ability bite food	1.1 (0.1)	1.1 (0.1)	1.0 (0.0)	1.0 (0.0)	1.0 (0.0)	4.2 (0.5)*#	1.6 (0.4)	3.0 (0.8)*
PCA, satisfied ability chew food	1.1 (0.1)	1.1 (0.1)	1.0 (0.0)	1.0 (0.0)	1.0 (0.0)	4.1 (0.7)*#	1.7 (0.5)	2.7 (0.7)*
OBC	22.4 (4.0)	NA	29.0 (2.5)	NA	20.0 (2.0)	NA	23.3 (4.5)	NA
PHQ–9	3.7 (0.9)	NA	4.9 (1.1)	NA	3.0 (1.0)	NA	5.4 (1.7)	NA
PSQI	5.4 (0.9)	NA	6.6 (0.1)	NA	4.0 (0.5)	NA	4.5 (0.7)	NA
Gad–7	4.0 (1.5)	NA	5.0 (1.8)	NA	2.8 (0.1)	NA	4.4 (1.2)	NA

(*)In the same row indicates significant higher within-group differences (Baseline and after injection and OMT) considering bruxer and control participants that received IS or NGF following pairwise post hoc comparisons (*P* < 0.01).

(#)The same column indicates significant between‐group differences following pairwise post hoc comparisons (*P* < 0.05).

*JFLS–20* Jaw function limitation scale–20, *PCA* Perceived chewing ability, *OBC* Oral behavior checklist, *PSQI* Pittsburgh sleep quality index, *GAD–7* Generalized anxiety disorders, *PHQ–9* Patient health questionnaire–9, *NRS* Numeric rating scale, *NA* Not applicable. Data are presented as mean and standard error (SE).

## Discussion

The current study is the first, to our knowledge, to show that bruxers (i.e., NGF_Bruxer and IS_Bruxer) did not significantly change the central modulation of motor pathways as a consequence of NGF-induced sensitization in combination with a 58-min motor training task. In contrast, significantly reduced and increased jaw-closing muscle corticomotor excitability and map area were identified after training in the control participants who respectively received NGF (i.e., NGF_Control) and IS (i.e., IS_Control) intramuscular injection (Figs. 1 and 2). MEP quantification in response to nociceptive inputs suggests that bruxers and controls probably are driven by distinct central regulations of the corticomotor pathways^11,13–15^. Indeed, the MEP amplitude related to FDI, i.e., the control hand muscle, did not significantly change. Based on that, and in accordance with previous studies, the FDI–MEP amplitude represents an internal control in the study design assessing cortical excitability of jaw-closing muscles^11,16^. Therefore, our results confirmed the raised research hypothesis since the masseter corticomotor excitability changed in controls, but not in bruxers in response to nociceptive inputs and a training-induced effect.

The results of the current study agree with previous evidence indicating that NGF nociceptive input in control participants significantly suppresses increases in motor excitability as a consequence of NGF-sensitization^5,10^ and also agree with a previous study reporting that repeated jaw movements can trigger short-term neuroplastic changes in the corticomotor control of jaw-closing muscles in healthy individuals, but to a lesser extent in bruxers who performs the repetitive tooth clenching or grinding^11^. Bruxers may require less brain activity to execute the same jaw movements compared to individuals without bruxism, which may reflect that neuroplastic changes may already have occurred in bruxers, and there is no functional need for the central nervous system to react to the specific task^11^. It is consistent with studies reporting differences in the neural pathways related to the corticomotor control of the jaw-closing muscles of bruxers and non-bruxers^11,13,14^ and healthy individuals, before and after a TCT^11,17–19^. Metaplasticity may also impact corticomotor excitability in bruxers as a result of a series of time-dependent events. It refers to the mechanism for maintaining the overall synaptic weight of a neuronal network in the physiological range, which may provide distinct corticomotor excitability in controls and bruxers. Indeed, since changes are learning-specific, they may play a role in the underlying mechanisms of long-term potentiation considering memory and learning^20^. Based on this, we speculate possible no long-term effects of the intervention, mainly in control participants. Interestingly, although the NGF‐induced sensitization can significantly reduce jaw-closing muscle corticomotor excitability in controls, but not in bruxers, the higher self-reported masseter pain intensity and jaw function disability and EMG CV changes were positively associated with the injection of painful substances into the muscles, in both controls and bruxers. These findings are consistent with a recent study reporting that increased pain may be induced by intramuscular injection of NGF and glutamate in healthy humans due to increased expression of peripheral N-methyl-d-aspartate (NMDA) receptors^21^. It could be argued that although NGF-induced masseter muscle sensitization causes different levels of inhibitory corticomotor excitability in bruxers and controls, in both participants, the NGF-sensitization changed EMG activity. Again, this likely occurs to prevent further musculoskeletal damage, and it seems associated with lower pain intensity on function^1,10,22^. Based on that, the findings observed in this study did not support the so-called ‘vicious cycle’ concept^7^ in agreement with previous studies^4,10,23,24^. The inhibitory effect of muscle pain on motor function can be interpreted as an ‘adaptation’ to pain (i.e., Pain Adaptation Model Theory) in order to limit overall movements aiming to protect the affected painful muscle area from further injury and, therefore, promote healing^9^.

In the pain field, a rapid effect of pain on cortical motor plasticity has been observed in response to acute and chronic pain^25^, which may impede training-induced functional neuroplasticity manifested as decreased corticomotor excitability as defined by TMS^26^. Sensory-motor integration at a reflex such as a motor withdrawal reflex in response to noxious stimuli acts as a protective response to noxious stimulation^25^. In these cases, changes in synaptic inputs may alter cortical mapping, and the inhibition of the motor cortex by pain may be the underpinning of the evolution of pain as a result of dysfunctional circuits and loss of neurons within circuits known to be present in chronic pain^25^. Therefore, as clinical consequences, a central point in the discussion of our findings is related to the better understanding of bruxism physiology and pathophysiology, and myofascial pain mechanisms. It could be speculated that pain caused by NGF-induced sensitization could help bruxers unlearn or “detrain” repetitive masticatory muscle activity, changing their oral behavior^11^. Indeed, our findings may have implications in motor learning and performance understanding, with a putative impact on rehabilitative procedures such as physiotherapy^26^. Since everyone has potential biological prerequisites and resources developed through the course of life our findings providing a better knowledge on orofacial pain and function and its assessment and strategies for management^8^.” The effect of pain on jaw-closing muscle performance has been a subject of interest in earlier experimental studies^10,23^, applying or not methods to distinguish bruxers or non-bruxers participants. In the current study, the accuracy and precision of the MVC were positively associated with series, target force levels, and groups, considering the experimental pain model, training-induced effect, and specific criteria for characterization of definitive sleep bruxism. A previous study assessing MVC performance between definitive sleep bruxers and control participants revealed that accuracy was significantly dependent on the series and target force level; at the same time, no significant differences between groups were observed in the jaw movement precision in terms of training-induced effect^11^. Our results were also significantly dependent on the series and target force level. Overall, groups showed significantly higher accuracy and precision in the second (i.e., with feedback) than in the first and third series. These results agree with early reports demonstrating that the observation of jaw movements to reach a specific target position was associated with more accurate and precise performance than when no visual observation was provided^11,23,24^. Yet, different from the Ikuta et al.^11^ study, we have also identified differences among groups not only for accuracy but also for precision. The contradictory findings related to differences among participants can, at least in part, be explained by a possible influence of NGF-induced sensitization on MVC performance, which was not used in the previous study.

Significant between-group differences in the precision of jaw movements were observed at 10% MVC, with the NGF_Bruxer and NGF_Control groups showing a significantly lower CV (i.e., higher precision MVC) in the first series. It suggests that muscular pain modulated by NGF-induced sensitization, even at a low-force level, has modulated the training performance, thus influencing the precision of the jaw movements among participants who received NGF nociceptive sensitization. It probably occurs because muscle pain may increase with stronger contractions. Thus, the painful muscle would serve as muscle control for maintaining a constant EMG level of contraction^23^. However, motor performance can also reflect other functional changes, not necessarily only neuroplastic ones^24^.

In terms of the MVC accuracy, differences between participants identified that controls who received NGF (i.e., NGF_Control) showed a significantly lower relative error, i.e., higher accuracy, at middle TCT level (20% MVC) in the third series, and bruxers with a sensitized jaw muscle (i.e., NGF_Bruxer) the higher relative error, even at the lowest TCT level (10% MVC). However, it suggests that only such peripheral changes may not easily explain the present data. More likely, central neuroplasticity plays a more important role in the jaw movement performance observed for bruxers and controls. The use of an experimental muscle pain model that yields muscle sensitivity, and distinct discrimination between bruxers or controls, can partially explain the differences in our findings when compared to previous evidence of no NGF‐induced changes in the masseter corticomotor excitability in healthy participants^11,23^. Notably, the specific neural pathways of bruxers, in combination with training exercises and a pain model, can increase the relative error of MVC (Fig. 4). Again, these findings not only indicate that NGF‐sensitized muscles may decrease corticomotor excitability differently in control participants but also suggest that the jaw pain intensity is probably driven by each one specific control neural protective mechanisms, which likely occurs to prevent further damage.

Some study strengths need to be addressed: (a) we have also assessed a control site (hand muscles) to measure motor cortex excitability induced by training and nociceptive inputs^11,16^; (b) we have used specific criteria for discrimination between definitive bruxers and controls, avoiding only self-reported criteria for bruxism assessment; (c) a biting device was used to ensure the standardized masseter background contraction across participants^17^; (d) we have conducted a randomized, double-blinded (examiner and participant) placebo-controlled study, which precludes biased inferences and results; and (e) finally, we have included exactly the number of participants estimated on sample size calculation, which in turn assured an adequate power to detect significantly within‐between interactions. Thus a minimum number of participants was included, which is a critical ethical aspect in the design of a planned research protocol since avoiding an unnecessarily exposing more subjects to the protocols tested respecting ethical criteria, costs, and burdens of a clinical trial^27^. However, this study is not free of limitations. We should be cautious for inferring about bruxism and pain as the pain was not an inclusion criterion for participants in this study; nevertheless, we have used a well-known and validated experimental pain model to ensure standardization across subjects^5,10,27^. In addition, due to the high variation of the individual reactions to jaw muscle pain and tooth clenching and grinding, the extrapolation of our results to the population should be done with caution. Still, this study had a short follow-up. Neverthelles, the NGF-induced masseter hyperalgesia can last for up to 2 weeks^10^; thus, the effect of NGF masseter sensitization in combination with standardized training might be less variable and easier to assess within a short follow-up time. Therefore, although the information about the long-term follow-up after the last training session could have been interesting, it will await further studies. Moreover, this variation could explain why some patients develop chronic pain while others do not^28^. Further studies are needed to investigate the effect of bruxism physiology and pathophysiology according to the circadian cycle since considerable variability in terms of classification of bruxism and assessment of neuroplasticity hamper a definite conclusion^15^. Finally, further studies should also be conducted in order to establish how long the neuroplasticity lasted to provide important insights into the time-course of corticomotor neuroplasticity related to NGF-nociceptive inputs and tooth clenching training exploring in more detail the temporal profile of the sensitization effects.

## Conclusion

NGF‐induced masseter muscle soreness in combination with a training-induced effect can significantly reduce jaw-closing muscle corticomotor excitability and substantiates the occurrence of significant central changes in control, but interestingly, bruxers do not show similar degrees of modification. In turn, NGF sensitization had some impact on force control mechanisms and masseter muscle performance and positively influences the occurrence of significant higher jaw pain intensity and limitation, in both bruxers and controls, that most likely aim to protect the musculoskeletal orofacial structures. Further studies with larger sample sizes will be needed to test new concepts of de-training in bruxers.

## Methods

### Study design, ethical aspects and participants

**Figure 5:**
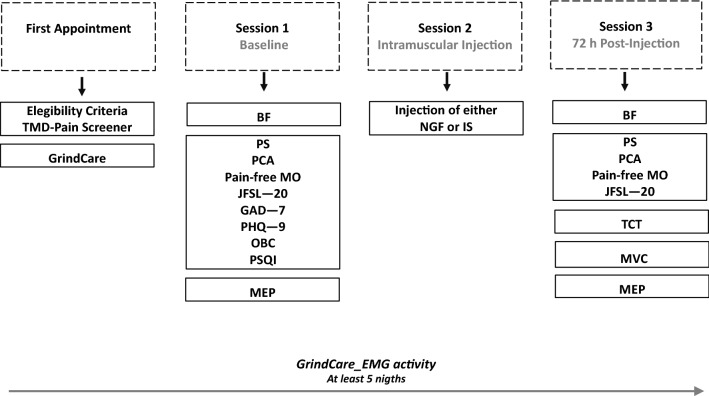
Study timeline: *(i) First appointment*, participants were interviewed about eligibility criteria, adequately instructed and trained for using the electromyographic (EMG) device (GrindCare) during sleep for at least five consecutive nights during the experiment, i.e., at least 48 h before and 72 h post-either NGF or IS injection administration, and answered the TMD—Pain Screener; *(ii) Session 1*, motor evoked potential (MEP), bite force (BF) and pain‐free mouth opening (MO) were assessed, and also participants answered questions about jaw pain and function, i.e., Pain Scale (PS), Perceived Chewing Ability (PCA), Jaw Function Limitation Scale—20 (JFSL—20), Generalized Anxiety Disorders (GAD—7), Patient Health Questionnaire—9 (PHQ—9), Oral Behavior Checklist (OBC), and Pittsburgh Sleep Quality Index (PSQI); *(iii) Session 2* (24 h after session 1), participants received either injection of nerve growth factor (NFG) or isotonic saline (IS, control) in the right masseter muscle randomly assigned; *(iv) Session 3,* (72 h post-either NGF or IS injection), participants performed a tooth clenching task (TCT) of three series in a randomized order for a total of 58 min, and again, MEP, BF, maximum voluntary contraction (MVC) and pain‐free MO were assessed and participants answered the PS, JFLS—20 and PCA questionaires.

**Figure 6:**
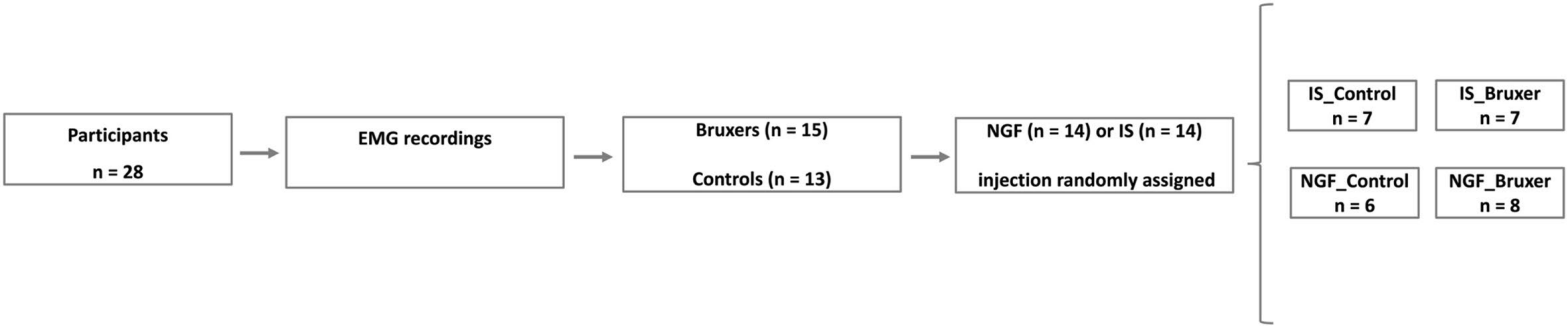
Characterization of participants as bruxers or controls based on EMG activity recorded at least 48 h before any intervention and after receiving either injection of NFG or IS in the right masseter muscle randomly assigned in combination with a TCT. Tooth clenching task (TCT); nerve growth factor (NGF); isotonic saline (IS); electromyographic (EMG).

This randomized, double-blinded placebo-controlled study used an experimental pain/sensitization model caused by intramuscular NGF administration in combination with a standardized TCT. The study timeline comprises the *first appointment* with eligibility criteria interview and instruction for using a single-channel EMG device (GrindCare) for classification of participants as definitive bruxers or controls (non-bruxers)*; session 1*, i.e., baseline; *session 2*, i.e., all participants classified as bruxers or controls randomly received either an injection of NFG or IS into the right masseter muscle; *session 3*, i.e., 72 h post-injection in combination with a TCT as shown in Fig. 5. It resulted in a study design with 4 arms: IS_Control (i.e., Control participants who received IS injection); IS_Bruxer (i.e., Bruxer who received IS injection); NGF_Control (i.e., Control participants who received NGF injection); and NGF_Bruxer (i.e., Bruxer who received NGF injection) as illustrated in Fig. 6. Participants were also asked to score the intensity of clinical symptoms related to their jaw muscles, defined as pain and function, fill out questionnaires regarding psychological traits and sleep quality, and report tooth clenching or grinding at night. The following outcome variables were assessed at two-time points, i.e., at baseline and 72 h post‐either NGF or IS injection in combination with a TCT: (a) right masseter muscle MEP amplitude (primary outcome); (b) accuracy and precision of jaw movements; and (c) clinical assessment of jaw pain intensity and function (secondary outcomes). The (d) psychological and sleep conditions were assessed only at baseline.

The study, including all experimental protocols, was approved by the Central Denmark Region Ethics Committee (approval number: 1-10-72-47-20, Aarhus, Denmark) and conducted in accordance with the Helsinki Declaration II, relevant guidelines and regulations. All participants who agreed to participate in the study gave their voluntary written informed consent prior to the experiment participation. The clinical trial was registered in The Brazilian Clinical Trials Registry (ReBEC, number: RBR-2wcyvss, on 20/04/2022).

**Figure 7:**
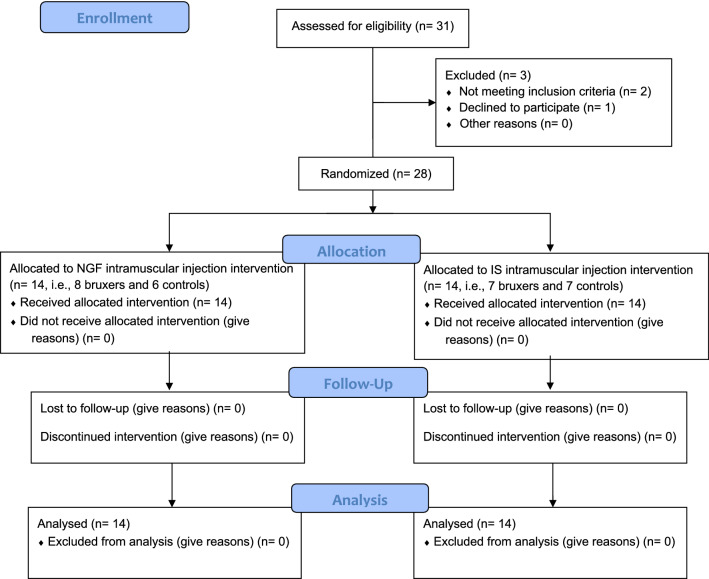
Flow diagram depicting allocation process.

Thirty-one subjects were assessed for eligibility, 3 were excluded: 2 because not meeting inclusion criteria, and 1 declined to participate in the study (Fig. 7). Finally, a total of 28 participants (mean age: 24.1 ± 3.6 years, age range: 18–30, 9 men), with no significant between‐group differences in the sex distribution (*P* = 0.260) and age (*P* = 0.976), were voluntarily recruited by advertising posted inside the campus of Aarhus University and on a webpage of the Section for Orofacial Pain and Jaw Function (http://odont.au.dk/om-odontologi/sektioner/kof/), and invited to participate in the study. The volunteers participating in the study were in good general health, aged > 18 years, and with no orofacial pain complaints in the last 30 days or chronic pain disorders (i.e., pain‐related TMD symptoms were ruled out with the aid of the TMD‐pain screener) ^29,30^. Presence of dental or medical illness; regular intake of medication such as antidepressants, anticonvulsants, or nonsteroidal anti‐inflammatories, any diagnosis of psychiatric or personality disorders, and the presence of contraindications to TMS (i.e., metal implants in the head, implanted electronic devices, history of epilepsy and if they were pregnant) were defined as exclusion criteria^31–33^. All assessments and experimental procedures were conducted at the Department of Dentistry and Oral Health, Aarhus University.

Sample size calculation was estimated considering 5% as α error, 20% as β error, 2.0 μV as standard deviation and a minimum detectable mean difference of 5.8 μV in the corticomotor excitability according to masseter sensitization due to treatment effect based on a previous study^10^. According to randomly assigned NGF (n = 12) or IS (n = 12) masseter intramuscular injection, a total of 28 participants were estimated to compose the total sample size, which were allocated into 4 groups according to bruxers or controls characterization: IS_Control (n = 7), IS_Bruxer (n = 7); NGF_Control (n = 6), and NGF_Bruxer (n = 8). There were no losses and exclusion of participants after intramuscular injection randomization and characterization as bruxers or controls, and the number of participants per group was sufficient to detect significant corticomotor excitability differences related to experimental pain models and participants characterization.

For discrimination between definitive bruxer and control participants, the EMG activity of the temporalis muscles was recorded with the portable single-channel electromyographic (EMG) device (GrindCare, Medotech A/S, Herlev, Denmark). In the first appointment, participants were adequately instructed and trained to use the EMG device and asked to wear the device during sleep at home for at least five consecutive nights during the experiment, i.e., at least 48 h before and 72 h post-either NGF or IS injection administration. The electrode was placed at the anterior temporalis muscle, which provides the same information as EMG recordings from the masseter muscles during sleep^33^. If any participant had more than 4 recordings showing more than half of the sleeping hours with over 19 EMG events/hour or higher, they were in this study considered to be bruxers^11,34^.

Self-reported data on tooth clenching and grinding and clinical symptoms were also collected using criteria proposed by Lobbezoo and collaborators^3^ to identify the “possible bruxism” and “probable bruxism.” According to these criteria, the “possible sleep bruxism” is based on a positive self-report of the night tooth clenching and grinding, and the “probable sleep bruxism” is based on a positive clinical finding, with or without a positive self-report of the night tooth grinding or clenching. Therefore, participants were also asked about a positive self-report of the nighttime tooth clenching or grinding with the following questions: “Have you been told, or do you notice that you grind/clench your teeth or clench your jaw while sleeping at night” and also about clinical symptoms using the following questions: “Is your jaw ever fatigued or sore on awakening in the morning?”; “Are your teeth or gums ever sore on awakening in the morning?”; and “Do you ever experience temporal headaches on awakening in the morning?”.

### Experimental procedures

Participants were placed on a dental chair in a supine position supported by a headrest for MEP recordings. A flexible cap was placed over the head in a standardized way based on anatomical markers and in accordance with the International 10–20 Electrode Placement System guidelines^35^. EMG activities from the first dorsal interosseous (FDI) (control) and right side of the masseter muscles were prompted by the TMS using a Magstim 200 stimulator (Magstim 200, The Magstim Co. Ltd., Whitland, Dyfed, UK) and recorded using bipolar surface electrodes (Ambu, Neuroline 720, Copenhagen, Denmark). For FDI MEP recording, the bipolar surface electrodes were placed over the FDI (muscle belly–caput metatarsal I), and the figure-of-eight stimulating coil was oriented 45° obliquely to the sagittal midline to the left side of the scalp^36^. For masseter muscles, the surface electrodes were placed 10 mm apart and parallel to the main direction of the muscle fibers, along the central part of the masseter muscle (i.e., midway between the anterior and posterior borders determined by manual palpation) and the origin (inferior to the bony margin of the zygomatic process) and insertion (superior and anterior to the mandibular angle and lateral surface of the ramus of the mandible). The MEPs in the right masseter muscle were evoked by TMS of discrete areas of the left scalp, approximately 4 cm anterior to the Cz and 9 cm lateral to the mid-sagittal plane^37^. During the assessment of motor threshold (MT) and MEPs, the participants kept a special biting device between the anterior teeth to ensure a constant pre‐activation of the masseter, which is required to elicit masseter MEP, and to ensure standardization of background activation across subjects^17^. The biting device was previously calibrated to 10 N when the two parts were in contact, thus providing the participant with continuous feedback on the targeted force level allowing a constant and reproducible activation of the jaw‐closing muscles^11,17^. The display sensitivity of the traces was previously adjusted to 20–50 μV, which allows to distinguish the MEP from background EMG activity clearly discernible on the monitor from 12 consecutive stimuli^10^. For both muscles, the ground electrode was placed around the right wrist. The sampling rate was 4 kHz, and the EMG signals were amplified, filtered (10 Hz–3 kHz), and stored on Viking Select (Nicolet EDX, Natus Medical Inc., Pleasanton, CA, USA). The MT of the FDI and right masseter muscles was measured and defined as the minimum stimulus intensity that produced 5 out of 10 discrete MEPs clearly discernible from the background EMG activity in each muscle^19,23^.

The MEPs were assessed by stimulus–response (S–R) curves and motor cortex mapping as previously described^10,36^. S–R curves were constructed in steps of MT, from T–10% T + 20% to T + 60% (i.e., respectively at 90, 100, 120, and 160% MT), where MT was the resting or active MT measured at the specific time of creating the S–R curve. MEP amplitude (μV) was the average of 12 stimuli delivered at each stimulus level, with an interstimulus interval of 10–15 s. After S–R curves, eight TMS stimuli were delivered to each grid site over the scalp identified by the flexible silicone cap marked with the 1–1 cm^2^ grid in an anterior–posterior to create the corticomotor mapping and lateral–medial coordinate system. The anterior–posterior grid lines relate to the vertex in accordance with the 10–20 EEG electrode placement system, and the stimulator output was set at 20% (120%) above the MT (120%). The motor cortex areas (cm^2^) were calculated in accordance with a previous study^11,17^.

For intramuscular NGF or IS Injection administration, participants were randomly assigned by a computer-generated list (randomization.com) into two groups according to the type of injection that was applied into the right masseter muscle. A staff member, who was not involved in the eligibility evaluation or outcome assessments, prepared the solutions for the injections, randomization code, and allocation. Therefore, both the examiner and participants were blinded to the experimental conditions. For injection, the needle was perpendicularly inserted into the masseter body until bone contact was felt, after which the needle was slightly retracted (approximately 2 mm), aspiration was done, and the bolus injected in approximately 10 s. Procedures were done according to the type of injection that was administered into the right masseter: (a) experimental muscle pain group (NGF group, *n* = 14) where 5 µg NFG diluted in sterile water (0.2 ml) was injected after a negative aspiration test; or (b) control injection group (IS group, *n* = 14) where the participants received an injection of 0.2 ml of a sterile solution of IS (0.9%) after a negative aspiration test^10^.

The tooth clenching tasks (TCT) consisted of 58-min of a standardized TCT of three series according to previous studies^10,36^. The participants were seated comfortably on an office chair, and a U-shaped force meter (Aalborg University, Aalborg, Denmark) was used to measure the actual force value. First, participants were asked to perform a maximum tooth clench at their right first molar teeth on the force meter to define the 100% MVC. Following the maximum voluntary contraction (MVC) test, participants were instructed to clench their right first molar teeth on the force meter connected to a computer monitor and software (Force Calibration Analyzer, Denmark) in three series. Participants were simply instructed to perform jaw movements following different target force levels without a visual feedback in the first and third series. In the second series, a visual feedback of the muscle activity level via a force meter was displayed to the participants on a screen. Each serie consisted of 10, 20, and 40% of the MVC, and one measurement consisted of one force level (10, 20, or 40% of the MVC) in a randomized order. During all measurements, participants alternately performed a 30 s rest block and a 30 s task block for 360 s. In the task block, participants alternately performed a 5 s rest block and a 5 s task block. The coefficient of variation (CV) was calculated from the actual force value at each force level in each series to evaluate the precision at each target force level, while the relative error between actual force value actual at each force level in each series was used to assess accuracy^11^.

For assessing jaw pain and function, psychological traits, and sleep quality, the participants were asked to score the intensity of clinical symptoms in their jaw muscles, defined as pain and function, on 0–10 numeric rating scale (NRS) at baseline and 72 h post-injection. Care was taken to explain the different symptoms. The intensity of jaw pain at rest and evoked by chewing, considering each session was rated on a 0–10 NRS, where 0 means “no pain at all” and 10 means “the worst pain imaginable.” Pain‐free mouth opening (mm) and perceived chewing ability (PCA) were measured, respectively, according to the DC/TMD examination guidelines^30^. PCA questions were scored and summed using a 7‐point Likert scale, and higher scores indicated lower perceived chewing ability^38^. In addition, the following questionnaires were applied as described in previous studies: Jaw Function Limitation Scale–20 (JFLS–20)^39^, Oral Behavior Checklist (OBC)^40^, PSQI^41^, Generalized Anxiety Disorders–7 (GAD–7)^42^ and Patient Health Questionnaire–9 (PHQ–9)^43^.

### Statistics

This study assessed differences between bruxer and control participants before and after the experimental intervention using either NGF or IS injection following the TCT. The outcome variables are reported as means and standard error of the mean (SEM). Normal distribution was assessed with the aid of the Shapiro–Wilk test, and log10 transformations were applied for the continuous variables when the results were significant, considering an alpha level of 5% (*P* < 0.05). Age and sex differences between bruxers and controls were calculated with a t-test and a Chi-square test.

Repeated‐measures analysis of variance (RM ANOVA) was calculated to assess differences in the MEP amplitude considering between-subject factors, *groups—4 levels* (IS_Control, IS_Bruxer, NGF_Control, and NGF_Bruxer), and two within‐subject factors, *assessment time—2 levels* (Baseline and after TCT), and *intensity of stimulation—4 levels* (90, 100, 120 and 160% MT). Likewise, RM ANOVA calculated differences in the precision and accuracy of MVC (log10 transformed values) considering two within‐subject factors, *series—4 levels* (First, second, third, and target force level), and *MVC— 3 levels* (10, 20, and 40%). Differences in corticomotor mappings areas for masseter MEP amplitude at 120% MT were also calculated considering the *assessment time—2 levels* (Baseline and after TCT). When appropriate, post hoc analyses were performed using the Bonferroni test.

One-way analysis of variance (ANOVA) and paired t-test were applied to compare, respectively, between and within‐group differences in jaw pain and function, chewing ability, JFLS–20 scores, and pain‐free mouth opening (mm). For the OBC, PHQ–9, PSQI, and GAD–7 scores, between-groups differences were calculated only at baseline using one-way ANOVA.

Finally, between-group differences related to night-to-night variability in EMG activity considering the number of EMG events during sleep, and the coefficient of variation (CV) from the multiple night recordings (CV: SD/mean) considering between-group differences at baseline and between-group differences after either NGF or IS injection administration were calculated using Kruskal–Wallis, followed by the post hoc Dunn’s test when appropriate. *P* < 0.05 was considered significant.
